# Diverse migration patterns and seasonal habitat use of Stone’s sheep (*Ovis dalli stonei*)

**DOI:** 10.7717/peerj.15215

**Published:** 2023-06-16

**Authors:** Grace E. Enns, Bill Jex, Mark S. Boyce

**Affiliations:** 1 Department of Biological Sciences, University of Alberta, Edmonton, Alberta, Canada; 2 WSP Canada, Calgary, Alberta, Canada; 3 Fish & Wildlife Branch, British Columbia Ministry of Forests, Smithers, British Columbia, Canada

**Keywords:** Migration, Habitat use, Stone’s sheep, Wild sheep, Thinhorn sheep, Brownian bridge movement model, Connectivity, Corridor, *Ovis dalli*

## Abstract

We describe temporal and spatial patterns of seasonal space-use and migration by 16 GPS-collared Stone’s sheep (*Ovis dalli stonei*) from nine bands in the Cassiar Mountains of northern British Columbia, Canada. Our objectives were to identify the timing of spring and fall migrations, characterize summer and winter ranges, map and describe migration routes and use of stopover sites, and document altitudinal change across seasons. Our last objective was to assess individual migration strategies based on patterns of geographic migration, altitudinal migration, or residency. Median start and end dates of the spring migration were 12 and 17 Jun (range: 20 May to 05 Aug), and of the fall migration were 30 Aug and 22 Sep (range: 21 Aug to 07 Jan). The median area of winter and summer ranges for geographic migrants were 630.8 ha and 2,829.0 ha, respectively, with a broad range from about 233.6 to 10,196.2 ha. Individuals showed high fidelity to winter ranges over the limited duration of the study. The winter and summer ranges of most individuals (*n* = 15) were at moderate to high elevations with a median summer elevation of 1,709 m (1,563–1,827 m) and 1,673 m (1,478–1,751 m) that varied <150 m between ranges. Almost all collared females (*n* = 14) exhibited changes in elevation use that coincide with abbreviated altitudinal migration. Specifically, these females descended to lower spring elevations from their winter range (Δ > 150 m), and then gradually moved up to higher-elevation summer ranges (Δ > 150 m). In the fall, they descended to lower elevations (Δ > 100 m) before returning to their higher winter ranges. The median distance travelled along geographic migration routes was 16.3 km (range: 7.6–47.4 km). During the spring migration, most geographic migrants (*n* = 8) used at least one stopover site (media*n* = 1.5, range: 0–4), while almost all migrants (*n* = 11) used stopover sites more frequently in the fall (media*n* = 2.5, range: 0–6). Of the 13 migratory individuals that had at least one other collared individual in their band, most migrated at about the same time, occupied the same summer and winter ranges, used similar migration routes and stopover sites, and exhibited the same migration strategy. We found collared females exhibited four different migration strategies which mostly varied across bands. Migration strategies included long-distance geographic migrants (*n* = 5), short-distance geographic migrants (*n* = 5), vacillating migrants (*n* = 2), and abbreviated altitudinal migrants (*n* = 4). Different migratory strategies occurred within one band where one collared individual migrated and two did not. We conclude that female Stone’s sheep in the Cassiar Mountains displayed a diverse assemblage of seasonal habitat use and migratory behaviors. By delineating seasonal ranges, migration routes and stopover sites, we identify potential areas of priority that can help inform land-use planning and preserve the native migrations of Stone’s sheep in the region.

## Introduction

Migration is common across ungulates and has evolved in response to spatial and temporal variation of resources (Fryxell & Sinclair, 1988; Berger, 2004). Migration is fundamental to the demographics of migratory ungulate populations (Fryxell & Sinclair, 1988; Berger, 2004), ecosystem processes, and nutrient dynamics (Helfield & Naiman, 2001; Holdo et al., 2007). However, ungulate migrations are declining and disappearing worldwide (Berger, 2004; Bolger et al., 2008; Harris et al., 2009). This loss of migratory behavior is often attributed to disturbances such as habitat loss on seasonal ranges (Harris et al., 2009), barriers that impede migratory movements (*e.g.*, highways, fences; Epps et al., 2005; Sawyer et al., 2013; Seidler et al., 2015), overhunting (Milner-Gulland et al., 2001), and climate change (Middleton et al., 2013; Aikens et al., 2020). To maintain migratory ungulates in North America, migration routes and seasonal ranges used by ungulate populations must be conserved if migratory ungulates are to continue to experience high levels of human disturbance (Berger, 2004; Thirgood et al., 2004; Harris et al., 2009).

**Figure 1:**
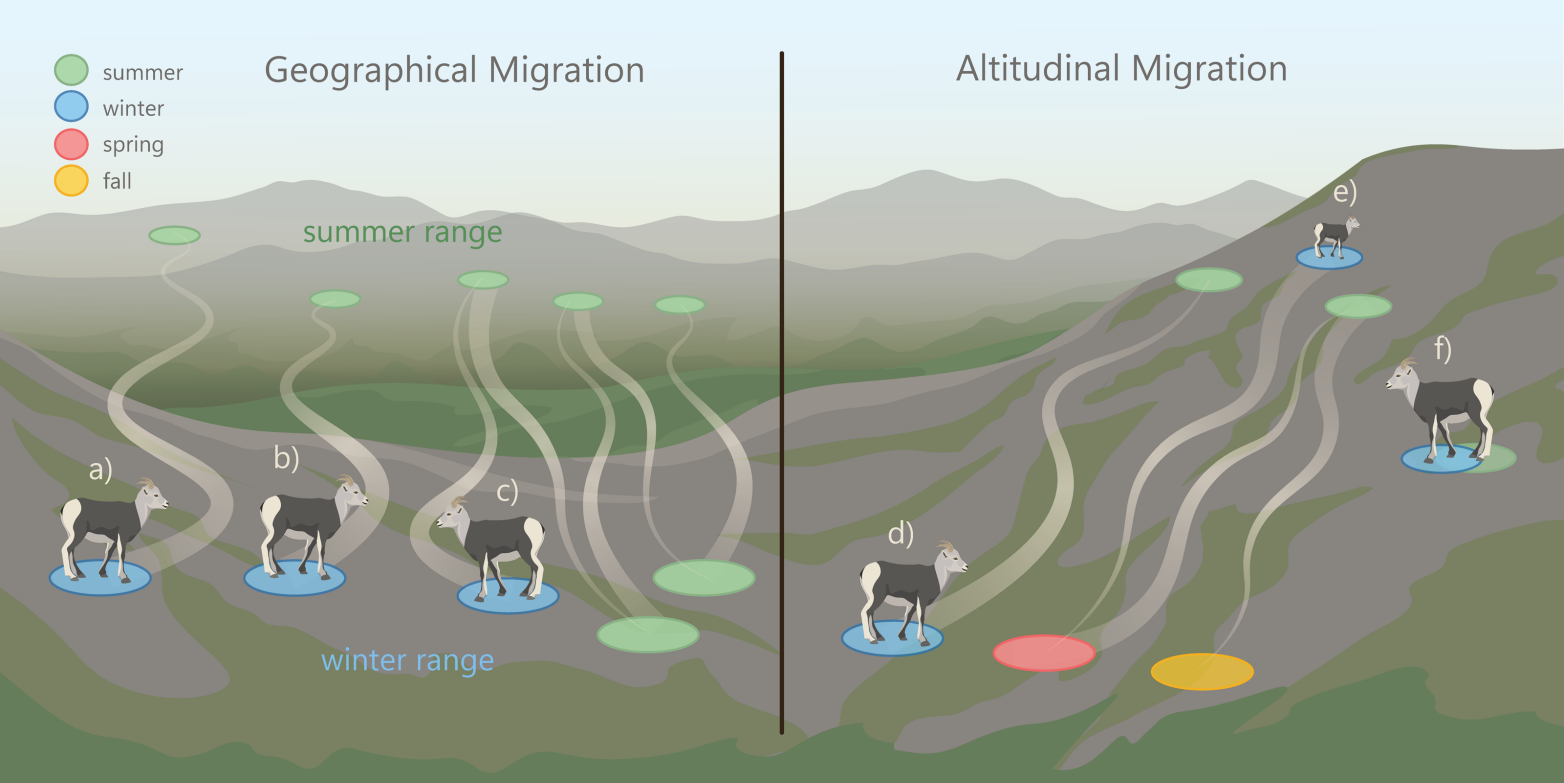
Patterns of geographic and altitudinal migration of wild sheep, exhibited by the collared female Stone’s sheep in the Cassiar Mountains, British Columbia, Canada. Geographic migration in this study was defined as movement away from a winter range to a geographically distinct summer range. We considered three types of geographic migration: a: *long-distance migration*, where sheep migrated > 20 km; b: *short-distance migration* where sheep migrated ≤ 20 km; and c: *vacillating migration*, where migrants occupy a spatially distinct range for most of the summer but vacillated (‘returned’) to their winter range ≥ 2 times (Denryter, Stephenson & Monteith, 2021). Altitudinal migration is the movement across an elevational gradient to seasonal ranges. We considered three types of altitudinal migration: d: *traditional altitudinal migration*, where sheep occupy low-elevation winter ranges and migrate to high-elevation summer ranges; e: *abbreviated altitudinal migration*, where sheep occupy high-elevation ranges in winter and summer and use lower elevations in the spring and fall (Courtemanch et al., 2017); and f: *residency*, where sheep remain on the same range year-round.Image credit: Fuse Consulting Ltd.

Mountain ungulates migrate geographically (over horizontal planes) and altitudinally (over vertical planes) to distinct seasonal ranges (Hebert, 1973; Albon & Langvatn, 1992). In the spring, mountain ungulates often “surf the green wave”, where they migrate in synchrony with the onset of new vegetation growth to access high-quality forage (Fryxell, 1991; Van der Graaf et al., 2006; Hebblewhite, Merrill & McDermid, 2008; Merkle et al., 2016). Ungulates also migrate to access other resources (*e.g.*, mineral licks; Heimer, 1973; Watts & Schemnitz, 1985; Ayotte et al., 2006; Ayotte, Parker & Gillingham, 2008) and to reduce predation risk (Fryxell & Sinclair, 1988; Hebblewhite & Merrill, 2007; Hebblewhite & Merrill, 2011; Sabal et al., 2021). North American ungulate migrations can vary extensively in their distance to reach seasonal ranges (Berger, 2004). For example, ∼7,000 elk (*Cervus elaphus*) in Wyoming were observed migrating 20 to 100 km from winter to summer ranges, while others remained on the same range year-round (Boyce, 1991). This herd also exhibited a seasonal change in elevation use that can be referred to as “traditional altitudinal migration”, where they occupied low-elevation winter and high elevation summer ranges (Fig. 1). In contrast to traditional altitudinal migration, some ungulate populations exhibit “abbreviated altitudinal migration”, where they occupy high elevation ranges in winter and summer and descend to lower elevations in the spring and fall (Fig. 1; Courtemanch et al., 2017). The elevations that mountain ungulates use in the winter are often influenced by snow distribution (Poole et al., 2016; Mahoney et al., 2018; Boelman et al., 2019), because ungulates typically avoid deep snow which reduces movement and access to forage (Parker, Robbins & Hanley, 1984; Dailey & Hobbs, 1989; Burles & Hoefs, 1984). Mountain ungulates that occur in regions where snow accumulates at low elevations often use windswept slopes with shallow snow cover located at high elevations (Poole et al., 2016; Courtemanch et al., 2017; Aycrigg et al., 2021).

Migratory diversity occurs when there is variation within a population’s migration strategies. Individual diversity in life histories, including migration, can improve population and ecosystem stability and resilience when faced with unexpected events (Webster et al., 2002; Morris et al., 2008; Schindler et al., 2010; Schindler, Armstrong & Reed, 2015; Griffiths et al., 2014). Despite the potential importance of migratory diversity, little research has examined the diversity among individual migration strategies in mountain ungulate populations (Lowrey et al., 2019; Lowrey et al., 2020). However, migratory diversity has been observed in both North American thinhorn (*Ovis dalli*) and bighorn sheep (*O. canadensis*) populations, where individuals migrate geographically and/or altitudinally to distinct seasonal ranges (Spitz et al., 2018; Lowrey et al., 2019; Lowrey et al., 2020). Some wild sheep populations exhibit partial migration (Lowrey et al., 2019; Lowrey et al., 2020; Spitz et al., 2018), where a portion of the population are migrants and a portion are residents (Fryxell & Sinclair, 1988). A novel migratory behavior has been described in the Sierra Nevada bighorn sheep population, termed ‘vacillating migration’, in which some individuals vacillated (travelled back and forth) between their winter and summer range during the winter (Fig. 1; Denryter, Stephenson & Monteith, 2021). Native bighorn sheep populations appear to have some of the longest migrations (30 to 50 km) when compared with translocated and augmented populations (Jesmer et al., 2018). Further, native populations of wild sheep exhibit the greatest amount of individual diversity in migratory behaviors compared to non-native populations (Lowrey et al., 2020).

**Figure 2:**
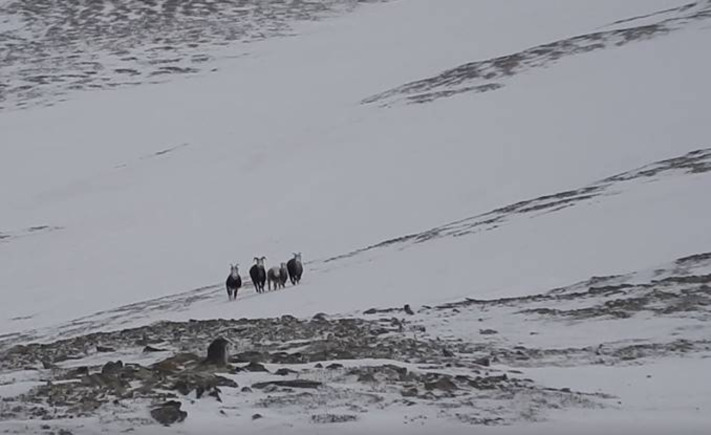
A band of five Stone’s sheep in the Cassiar Mountains with two adult females, two yearlings and one ram during capture efforts in 2018 taken from the ground (photo credit: Bill Jex).

Stone’s sheep (*O. d. stonei*) is a subspecies of thinhorn sheep found in northern British Columbia, Canada (Geist, 1971; Nichols & Bunnell, 1999; Sim et al., 2019). A native population of Stone’s sheep that was last estimated at 175 individuals in 2005 (B Jex, 2005, unpublished data) occurs in the Cassiar Mountains of northern interior British Columbia (Enns, Jex & Boyce, 2023). Within this population, small bands (groups) typically with five to 10 individual Stone’s sheep are dispersed throughout the mountain range (Fig. 2). Bands occupy distinct winter ranges and tend to only overlap the ranges of other bands during spring, summer, and sometimes the rut (mating season in late fall) (Enns, Jex & Boyce, 2023). Generally, female Stone’s sheep congregate and move with others in their band as one gregarious unit (Geist, 1971; Nichols & Bunnell, 1999). The native migratory behaviors and seasonal habitat use of the Cassiar Mountain population are expected to be largely intact, because anthropogenic activity is relatively localized and limited in the Cassiar Mountains. However, migratory behaviors and seasonal space-use in the Cassiar Mountain population, and in Stone’s sheep in general, is largely unknown.

The goal of our study was to document the seasonal habitat use and migratory behaviors of Stone’s sheep using telemetry data from GPS-collared female Stone’s sheep in the Cassiar Mountains. The first objective was to identify the timing of spring and fall geographic migrations. The second objective was to delineate summer and winter ranges and describe band composition and winter range fidelity. The third objective was to map and characterize migration routes and stopover sites. Our fourth objective was to document altitudinal change across seasons. Lastly, our fifth objective was to assess and classify the migration strategy of each collared individual as either a geographic migrant (long or short-distance), altitudinal migrant (traditional or abbreviated), vacillating migrant, or resident (Fig. 1).

## Materials & Methods

### Study area

**Figure 3:**
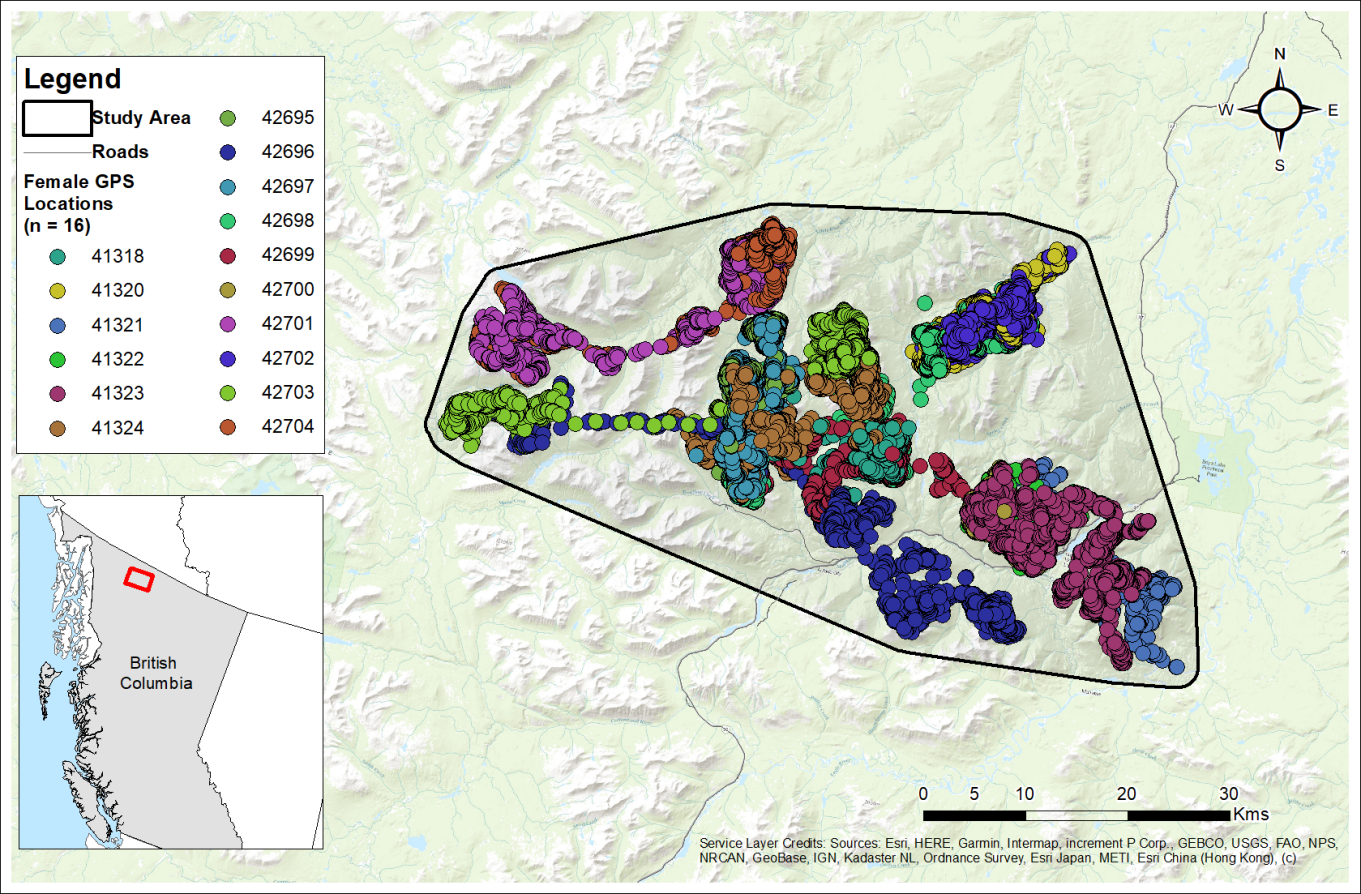
Study area and GPS collar locations of collared females (*n*= 16) from the Cassiar Mountain Stone’s sheep population in northern British Columbia, Canada, 2018–2020. Base Map provided by (Esri, Redlands, CA, USA).

The Cassiar Mountains span approximately 4,301 km^2^ of interior northwestern British Columbia, Canada (Pojar & MacKinnon, 2013) located near Cassiar, Jade City, and Good Hope Lake, within the Dease River and Kaska First Nations traditional territories. Our study area spanned 2,090 km^2^ in the north-central ranges of the Cassiar Mountains (Fig. 3). Most of the land in the Cassiar Mountains is managed through the Dease-Liard Sustainable Resource Management Plan (Government of British Columbia Ministry of Sustainable Resource Management, 2004) developed by the Province of British Columbia and local First Nations governments.

The Cassiar Mountains are within the Boreal Cordillera ecozone comprised of three ecosystems across different elevations (Meidinger & Pojar, 1991). At the lowest elevations (650–900 m) is the montane ecosystem, which consists of black spruce (*Picea glauca*), white spruce (*P. mariana*), Engelmann spruce (*P. englemannii*), lodgepole pine (*Pinus contorta*), trembling aspen (*Populus tremuloides*), dwarf birch (*Betula glandulosa*), and willow (*Salix* spp.). The subalpine ecosystem ranges from 900–1,500 m and contains white spruce, willow, dwarf birch in krummholz form, and subalpine fir (*Abies lasiocarpa*). Above treeline (>1,500 m) is the alpine ecosystem consisting of rocky, rugged terrain and plant communities comprised of few subalpine fir in krummholz form, grasses, sedges, alpine-flowering plants, lichens, and bryophytes (Meidinger & Pojar, 1991). Summer daylight periods extend beyond 18 h, with the longest days never becoming completely dark; core winter months provide less than 8 h of daylight, with much longer periods of extended darkness. Mean regional temperature during summer (1 June to 31 August) was calculated as 12.3 °C (SD = 3.1) and during winter (1 December to 31 March) as −12.1 °C (SD = 8.5) using daily temperatures recorded from 1998 to 2022 at the nearest Environment Climate Change Canada weather station located approximately 95 km from the study site in Dease Lake, BC (Government of Canada, 2023). Average annual precipitation in the Cassiar Mountains ranged from 460–700 mm and consisted of 35–60% snowfall (Geist, 1971; Wang et al., 2012).

Predators of Stone’s sheep in the Cassiar Mountains include golden eagles (*Aquila chrysaetos*), grizzly bears (*Ursus arctos*), black bears (*U. americanus*), wolves (*Canis lupus*), coyotes (*C. latrans*), wolverines (*Gulo gulo*) and lynx (*Lynx canadensis*) (Geist, 1971; Nichols & Bunnell, 1999; Koizumi & Derocher, 2019). Other ungulates include moose (*Alces alces*), mountain goats (*Oreamnos americanus*), caribou (*Rangifer tarandus*), and some elk and mule deer *(Odocoileus hemionus*).

Most of the Cassiar Mountains have remained unaltered by substantial anthropogenic disturbance because of low human densities and limited access to much of the area. Anthropogenic land use in the Cassiar Mountains includes vehicle traffic on Highway 37, several small jade mine operations in Troutline Creek and other nearby valleys, the abandoned Cassiar Asbestos Mine, gold placer-mining, hunting, and recreation including hiking, camping and off-road recreational vehicle use (snowmobiles, all-terrain vehicles; Jex et al., 2016). Although relatively low human disturbance, there is increasing interest in seasonal recreation, especially off-road vehicle-use and tourism, and potential for increased mineral exploration and extraction.

### Captures and GPS location data

We captured 18 adult female Stone’s sheep by helicopter net-gun in February 2018 and February and April in 2019. All individuals were collared with Iridium GPS collars (model G2110E2, Advanced Telemetry Systems, ATS, Isanti, MN, USA (Bertin, Single & Reflex, 2018) that collected GPS locations at 2 h (2018) and 1 h (2019) intervals. Capture and animal handling procedures were in accordance with BC Ministry of Forests, Lands, and Natural Resource Operations protocols, and approved by the Animal Care and Use Committee at the University of Alberta (AUP00002992).

Collaring efforts prioritized collaring females without a lamb-at-heal and from different bands to collect data that would represent movements across multiple bands in the Cassiar Mountains. As a result, the bands included in this study only had a subset of individuals collared.

GPS collars collected a total of 169,591 GPS locations from the time of captures until September 2020, unless an individual died before the end of the study, if their collar malfunctioned, or the collar batteries died. We removed erroneous GPS locations and locations that could have been influenced by capture effects by removing locations collected in the first week after captures (Werdel et al., 2021), locations collected with ≤ 2 satellites (251 locations), and locations with movement rates >20 km/h (2 locations) (D’Eon & Delparte, 2005; Lewis et al., 2007; Frair et al., 2010). We considered the time range of an individual’s location data to be adequate for evaluating an individual’s migration cycle if an individual’s location data extended at least 30 days before their spring migration to at least 90 days after their fall migration when they occupied their winter range. As a result, we removed data from two females that had <90 days of location data on their winter range after returning from fall migration. The remaining 16 females included in this study were dispersed across nine bands. For individuals for which data were collected for two full years in 2018 and 2019 (*n* = 4), and thus had two full migration cycles, we presented the findings on their migrations from 2019 only.

### Timing of geographic migrations

To identify females that exhibited geographic migrations (migrated horizontally to a distinct summer range), we identified individuals that exhibited an abrupt movement away from their winter range and did not return during summer months. To identify this abrupt movement, we plotted each individual’s net squared displacement (NSD) from their winter range in Migration Mapper (University of Wyoming, 2021) and R *v.* 4.0.3 (R Core Team, 2020). To calculate NSD, we determined a start date of which Euclidean distances were calculated from for each subsequent GPS location. We defined March 15 as the start date because we assumed Stone’s sheep would be residing on their winter range at that time. However, for individuals collared in early April 2019, we defined their start date as April 12, which was one week after the captures in April 2019. We assumed the individuals collared in April 2019 would still be occupying their winter ranges at this time.

Next, we plotted the GPS locations of each female alongside their NSD in Migration Mapper (University of Wyoming, 2021) and confirmed whether a female travelled away from her winter range to a spatially distinct (non-overlapping) summer range for at least 30 days during the summer (Eggeman et al., 2016). If a female used a spatially distinct summer range, she was considered a geographic migrant. Vacillating migration was described by Denryter, Stephenson & Monteith (2021) as occupying a spatially distinct range for most of the summer, but returning (‘vacillating’) to the winter range ≥ 2 times. Females that used a distinct summer range, but ‘vacillated’ between ranges at least twice in the summer based on their NSD and GPS location data, were identified as vacillating migrants. We identified the number of times vacillating migrants returned to the winter range during summer. Females that remained on the same range year-round, thus did not exhibit a geographic migration to a distinct summer range and did not vacillate between winter and summer ranges, were considered geographic residents.

We assessed each migrating female’s GPS locations and NSD to define the start and end dates of their spring migration as the date an individual moved away from their winter range and arrived on their summer range, respectively (Eggeman et al., 2016). Similarly, we defined the start and end dates of each female’s fall migration as the date an individual travelled away from their summer range and returned back to their winter range for at least 30 days (Eggeman et al., 2016). The winter and summer seasons for females that migrated were defined as the length of time between the end date of fall migration and the start date of spring migration, and the end of spring migration and start of fall migration, respectively.

We could not distinguish a winter and summer season for geographic residents, because geographic residents do not exhibit clear migrations to seasonal ranges. As a result, we based the summer and winter seasons for geographic residents on the timing of the spring and fall migrations of geographic migrants. The summer season for residents was defined as the time between the 66th percentile of spring migration end dates and the 33rd percentile of fall migrations start dates of the geographic migrants. Likewise, the winter season for residents was defined as the time between the 66th percentile of fall migration end dates and the 33rd percentile of spring migration start dates of the geographic migrants (Poole & Lamb, 2020, unpublished).

### Seasonal ranges

We developed summer and winter 95% utilization distributions (UDs) to represent each female’s summer and winter ranges by calculating Brownian bridge movement models (BBMM; Horne et al., 2007) at 50 m ×50 m resolution using the GPS locations from each female during their summer and winter seasons. We used the kernel Brownian bridge home range estimation function (Calenge, 2015) in R *v.* 4.0.3 (R Core Team, 2020). We defined the first smoothing parameter (*σ*1) using the R function *liker* (Horne et al., 2007), and the second (*σ*2) as 30 m (Frair et al., 2010; Kittle et al., 2015). We quantified the area of each female’s winter and summer ranges using the Calculate Geometry tool in ArcMap (Esri, Redlands, CA, USA). Next, we calculated the area and percent of overlap of a female’s summer range with their winter range using the intersect tool in ArcMap (Esri, Redlands, CA, USA). We provided the median and ranges for these summer and winter parameters separately for geographic migrants and geographic residents.

We assessed the amount of overlap between the winter ranges of all the females to determine which females were in the same bands. We considered females to be in the same band if their winter ranges overlapped >70% and if their telemetry locations showed them frequently moving together and occupying the same areas.

We assessed whether females exhibited fidelity to winter range in subsequent years by comparing the winter range used during the first year they were collared (2018 or 2019) to the winter range used in the following year (2019 or 2020). If a female’s consecutive winter ranges had >50% overlap, we determined that they expressed fidelity to their winter range. We had GPS locations over three winters for four females, and thus had three winter ranges with which we could assess winter-range fidelity. We determined that these four females expressed fidelity to their winter range if there was >50% overlap among all three years.

### Migration routes and stopover sites

We identified migration corridors and stopover sites used by migrating Stone’s sheep during spring and fall migrations by estimating BBMMs (Horne et al., 2007). Similar to the summer and winter ranges (UDs), we delineated 95% UDs for each spring and fall migration route at 50 m × 50 m resolution by estimating BBMMs (Horne et al., 2007) using the GPS locations from each female during their defined spring and fall migrations. In the BBMMs, we also included the locations collected 24 h before and after the start and end dates of the estimated migrations as suggested by Sawyer et al. (2009). As defined in the summer and winter BBMMs, we defined *σ*1 using the *liker* function (Horne et al., 2007) and *σ*2 as 30 m (Frair et al., 2010; Kittle et al., 2015). We estimated the distance travelled during spring and fall migrations for each female that migrated using the Calculate Geometry tool in ArcMap (Esri, Redlands, CA, USA). A combined migration corridor was generated and mapped for each band that had multiple collared migrants.

Next, we identified stopover sites located within the migration route UDs for each female. Stopover sites were defined as a spatial cluster of GPS telemetry locations collected over at least 12 h that were located along the migration route and not overlapping summer and winter ranges. We identified stopover sites as the top 10 percent of the UD from each migration corridor (University of Wyoming, 2021). We screened out stopover sites that had less than 12 h of location data per individual. If the nearest borders of adjacent stopover sites had a Euclidean distance <300 m, we combined the stopover sites into one stopover site using the reshape tool in ArcMap (Esri, Redlands, CA, USA). We presented the frequency of stopover sites used by individuals for both spring and fall migration and the population-level median and range.

### Altitudinal variation

To explore changes in the elevations used by females, we first summarized the median and range of elevations used by each female and the total collared population during the entire year, and during the summer and winter seasons. Elevations were extracted to each GPS location using the ‘amt’ package (Signer, Fieberg & Avgar, 2019) in R *v.* 4.0.3 (R Core Team, 2020) from the Canadian Digital Elevation Model (DEM) with 20 × 20 m resolution acquired from Natural Resources Canada (2020). Next, we calculated the change in elevations used between the winter and summer for each female (median winter elevation–median summer elevation) to identify if there was a substantial change in elevations used across seasons. Lastly, we explored each female’s elevation use throughout the year, by plotting mean daily elevations calculated over a 14-day moving window. A 14-day moving window was chosen to smooth out the ‘noise’ from minor changes in elevation that occurred daily. We used the plotted mean daily elevations for each female to identify general patterns in the elevations used during winter, spring, summer, and fall. We considered females that exhibited a change in mean elevation >250 m between the summer and winter seasons as exhibiting traditional altitudinal migration. Abbreviated altitudinal migration is defined by Courtemanch et al. (2017) as consisting of a spring altitudinal migration from low-elevation spring ranges to high-elevation summer ranges, followed by a descent to low-elevations in the fall and a return to high-elevation winter ranges (Fig. 1). We considered females that exhibited seasonal changes in elevation use that coincided with abbreviated altitudinal migration (Courtemanch et al., 2017) as abbreviated altitudinal migrants. Lastly, we identified females that did not exhibit traditional or abbreviated altitudinal migration, as altitudinal residents.

### Migration strategies

Classifying migration strategies used by mountain ungulates can be challenging because individuals often migrate over both horizontal (geographic migration) and vertical space (altitudinal migration). To simplify the classification of migration strategies we first classified individuals as geographic migrants or geographic residents. Geographic migrants were identified as individuals whose summer ranges overlapped <20% of their winter range for at least 30 days (Ball, Nordengren & Wallin, 2001; Eggeman et al., 2016). Geographic migrants were further classified as either long-distance migrants or short-distance migrants depending on whether the distance of their migration route was greater or less than 20 km, respectively. We chose 20 km as the cut-off for short-distance migrants because a natural break in the distribution of migration distances occurred at 20 km (Fig. S1). Any geographic migrant that exhibited vacillating migration (returned to their winter range ≥ 2 times in the summer) was reclassified as a vacillating migrant. Females that were identified as geographic residents (remaining on the same geographic range year-round) were further classified based on their seasonal changes in elevations used as a traditional altitudinal migrant, abbreviated altitudinal migrant or an altitudinal resident. Individuals that did not exhibit a geographic migration or altitudinal migration were classified as residents.

## Results

### Timing of geographic migrations

Geographic migrations from winter to summer ranges were demonstrated by 12 females. The median start and end dates of the spring migration were 12 June and 17 June (range: 20 May to 05 August; Table 1), respectively. During fall migration, the median start and end dates were 30 August and 22 September (range: 21 August to 07 January; Table 1), respectively. The length of time that females migrated in the spring and fall varied among individuals and bands. Generally, females from the same band migrated together or within a few days of one another (Table S1). The median duration of spring migration was 7 days, ranging from 1 to 26 days (Table S1). Fall migrations were typically longer with a median duration of 12 days and varied more across individuals than during spring migrations, ranging from 2 to 59 days (Table S1).

**Table 1:** Summaries of start and end dates of spring and fall migrations for female Stone’s sheep classified as geographic migrants in 2018 or 2019 (*n* = 12) in the Cassiar Mountains, BC, Canada. Summary information includes minimum, median, maximum dates and 33rd and 66th percentiles for spring and fall migrations.

**Migration dates**	**Min**	**33%**	**Median**	**66%**	**Max**
Spring start	20-May	05-Jun	12-Jun	15-Jun	10-Jul
Spring end	14-Jun	12-Jun	17-Jun	26-Jun	05-Aug
Fall start	21-Aug	18-Aug	30-Aug	19-Sept	11-Dec
Fall end	23-Aug	29-Aug	22-Sept	05-Oct	07-Jan

Females remained on their winter range for 67 to 211 days, with a median of 99.5 days. Like winter, the duration that females used their summer ranges varied across individuals from 43 to 183 days with a median of 69 days (Table S2).

**Figure 4:**
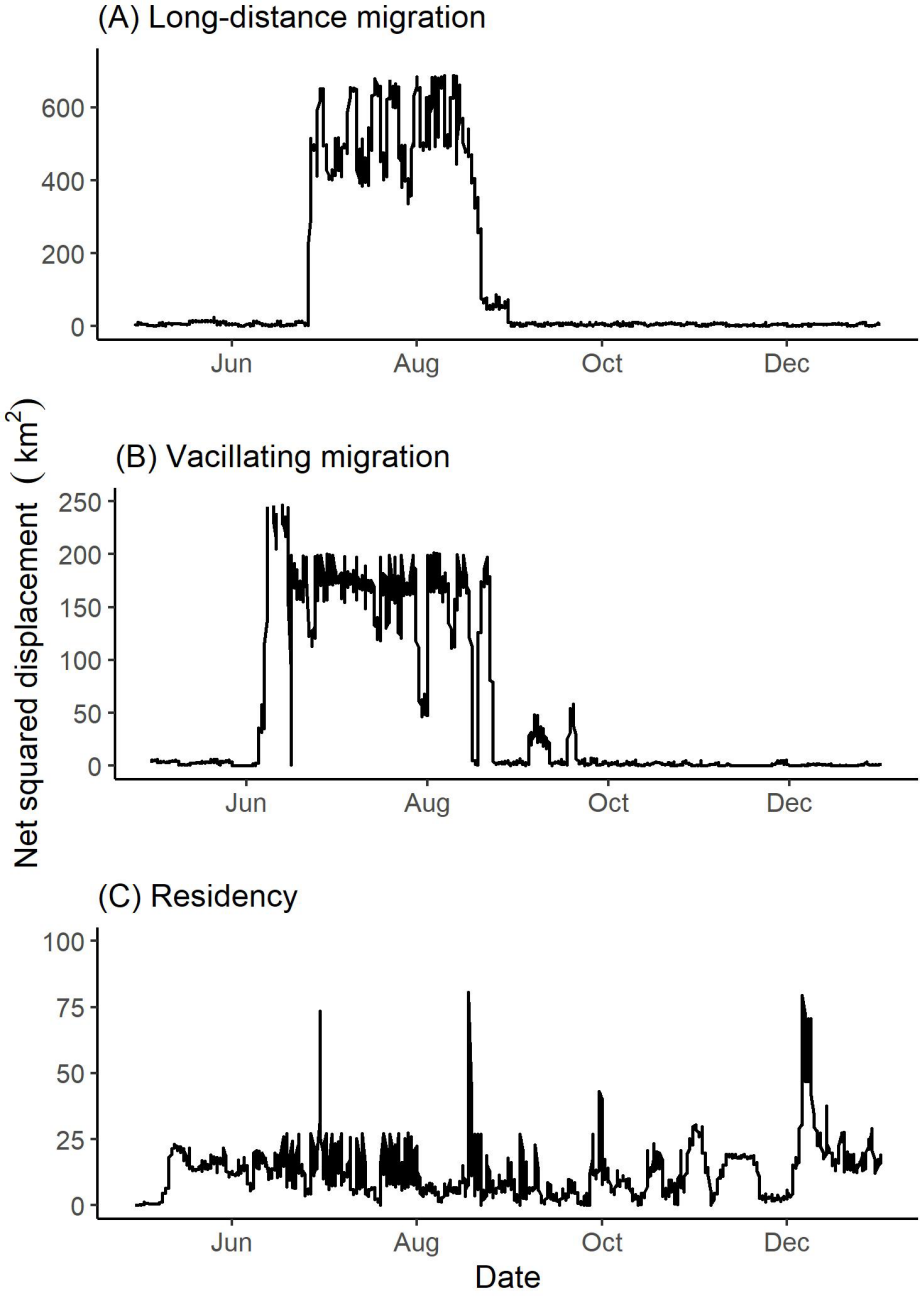
Examples of net squared displacement (km^2^) from the winter range exhibited by collared female Stone’s sheep exhibiting (A) long-distance migration (female 42704), (B) vacillating migration (female 41321), and (C) residency (female 41322). Net squared displacement was calculated from GPS collar locations of Stone’s sheep in the Cassiar Mountains, British Columbia, Canada, April 2019–January 2020. Ticks on the *x*-axis indicate the first day of the month.

Two females (from the same band) vacillated between winter and summer ranges (Table 2). These two vacillating females returned to the winter range three times during the summer season. Figure 4 depicts the vacillating behavior exhibited by one of the vacillating females that returned to winter range three times in the summer. We observed four females (from two bands) that did not migrate geographically to distinct seasonal ranges and thus were classified to be geographic residents (Table 2). Summer and winter seasons for residents were defined to be 26 June to 18 August and 05 October to 12 June, respectively (Table 1).

**Table 2:** Summary of geographic and altitudinal migration strategies by individuals and bands. Band number (No.; 1–9) order by descending migration distances (km), number of females in each band (n), study year, percent overlap (%) and area (ha) of summer on winter ranges, distance travelled (km) along geographic migration between winter and summer ranges, change in mean elevations (m; Δ elevation) from winter to summer, and the migration strategy exhibited by each collared female Stone’s sheep (*n* = 16) in the Cassiar Mountains, British Columbia, Canada, 2018–2020.

**Band No.**	***n***	**Female ID**	**Year**	**Area (ha)**			**Overlap (%)**	**Distance travelled (km)**	**Altitudinal variation** ^a^	**Migration strategy** ^b^	
				**Winter range**	**Summer range**	**Overlap**					
1	1	42696	2019	273.4	2887.8	0.0	0	47.4	−44.0	LDM	
										
2	1	42703	2019	610.8	1810.2	0.0	0	41.0	−31.6	LDM	
										
3	2	42704	2019	650.8	2476.3	0.0	0	31.3	−257.1	LDM	
		42701	2019	1195.1	2525.4	0.0	0	30.2	−7.7	LDM	
										
4	3	42698	2019	2308.0	10196.2	0.0	0	26.5	−110.2	LDM	
		41320	2019	1750.1	2732.5	516.3	29.5	–	−121.7	ABR	
		42702	2019	392.6	2453.7	181.0	46.1	–	−45.1	ABR	
										
5	1	41324	2019	277.9	4925.8	0.0	0	17.3	−6.8	LDM	
										
6	2	41318	2019	598.5	2459.2	15.0	2.5	15.2	−43.4	LDM	
		42699	2019	732.5	3598.0	9.5	1.3	14.0	21.4	LDM	
										
7	2	41321	2018	1852.9	6324.1	163.1	8.8	11.4	62.9	V-SDM	
		41323	2018	1738.8	3191.5	0.0	0	10.7	−44.3	V-SDM	
										
8	2	42697	2019	234.5	2770.2	19.9	8.5	7.9	4.9	SDM	
		42695	2019	233.6	2325.6	22.7	9.7	7.6	6.6	SDM	
										
9	2	41322	2019	1559.5	3237.7	506.8	32.5	–	7.5	ABR	
		42700	2019	1521.7	1765.5	1113.9	73.2	–	−22.3	ABR	

**Notes.**

a Altitudinal variation was calculated as the difference in median elevation used in winter versus summer (see Table S4). A negative value indicates use of a higher median elevation in the summer compared to winter.

b Migration strategies observed included, long-distance migration (LDM), short-distance migration (SDM), vacillating short-distance migration (V-SDM), and abbreviated altitudinal migration (ABR).

– not applicable

### Seasonal ranges

The median area of summer ranges for all geographic migrants (*n* = 12) was 2,829.0 ha and ranged from 1,810.2 ha to 10,196.2 ha (Table 2). Geographic migrants had much smaller winter ranges than summer ranges, where the median area of winter ranges was 630.8 ha and ranged from 233.6 ha to 2,308.0 ha. The amount of overlap between summer and winter ranges of geographic migrants was limited with a median of 0% ranging from 0% to 9.7% (Table 2).

Nine bands were identified from the 16 collared females in this analysis based on the overlap of winter ranges. The median percent of overlap of winter ranges within bands was 74% and ranged from 71% to 85% (Table 2). Most bands included two collared females. However, there were two bands with only one collared individual, and one band with three collared individuals. All bands were visually confirmed in the field to have more individuals than the number of collared individuals.

All 16 females (across the nine bands) returned to the same winter range that they had been collared the previous year, resulting in 100% fidelity to their winter range. Additionally, all females with three years of collar data (*n* = 4), returned to the same winter range in all three years.

Almost all females within the same band (*n* = 13 from six bands) used the same winter and summer ranges. However, one band with three collared females exhibited partial migration; where one female exhibited a long-distance migration to a distinct summer range, and the other two females were residents remaining in the same range year-round.

The median area of the winter and summer ranges for geographic residents (but later identified as altitudinal migrants) were 1,540.6 ha (range: 392.6–1,750.1 ha) and 2,593.1 ha (1,765.5–3,237.7 ha), respectively. The amount of overlap between summer and winter ranges of residents varied across individuals from 29.5% to 73.2% overlap with a median of 39.3% (Table 2).

### Migration routes and stopover sites

**Figure 5:**
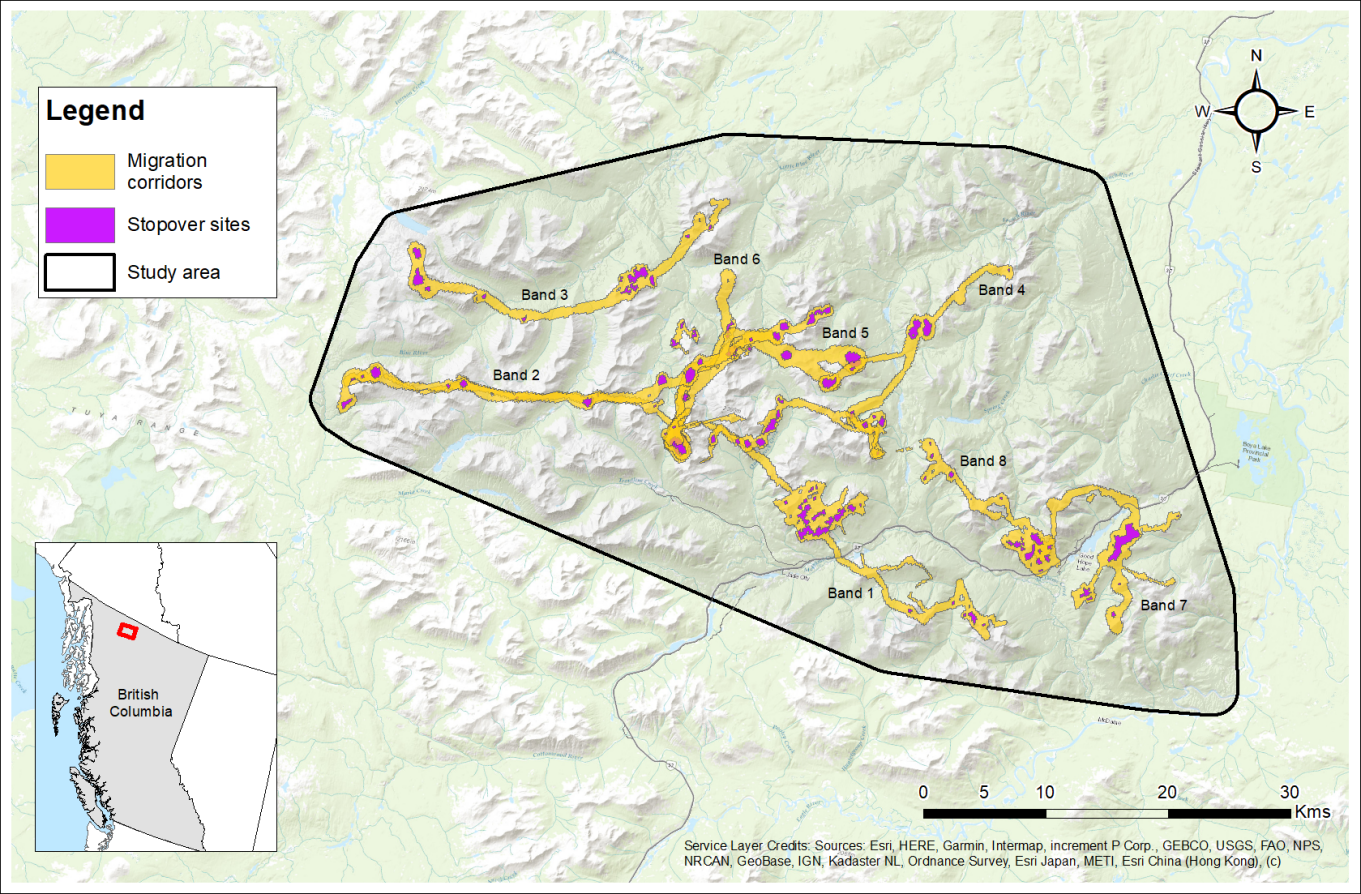
Migration corridors and stopover sites used during spring and fall migrations by female Stone’s sheep migrants (*n*= 12) in the Cassiar Mountains, British Columbia, Canada, 2018–2019. Migration corridors were estimated as utilization distributions using Brownian bridge movement models using GPS location data during spring and fall migrations. Stopover sites were estimated as the top 10 percent of each migration corridor utilization distribution. Bands (No. 1 to 8) are labelled with their corresponding migration routes. Base Map provided by (Esri, Redlands, CA, USA).

Females (and bands) varied in the distances travelled along their migration routes between winter and summer ranges (Table 2). The median distance travelled was 16.3 km ranging from 7.6 km up to 47.4 km (Table 2). Figure 5 shows the delineated migration corridors used by geographic migrants (*n* = 12) from eight different bands. We observed variation across individuals and bands in their use of stopover sites while on their spring and/or fall migrations (Fig. 5). During the spring migration, eight of the 12 migrants from four bands used at least one stopover site with a median of 1.5 stopover sites used ranging from 0 to 4 stopover sites (Fig. 5; Table S1). During the fall migration, 11 of the 12 migrants from eight bands used at least 1 to 6 stopover sites with a median of 2.5 stopover sites used (Fig. 5; Table S1). Geographic migrants within the same band typically used the same stopover sites, while migrants from different bands showed differences in the number of stopover sites used during spring and fall migrations (Table S1).

### Altitudinal variation

**Figure 6:**
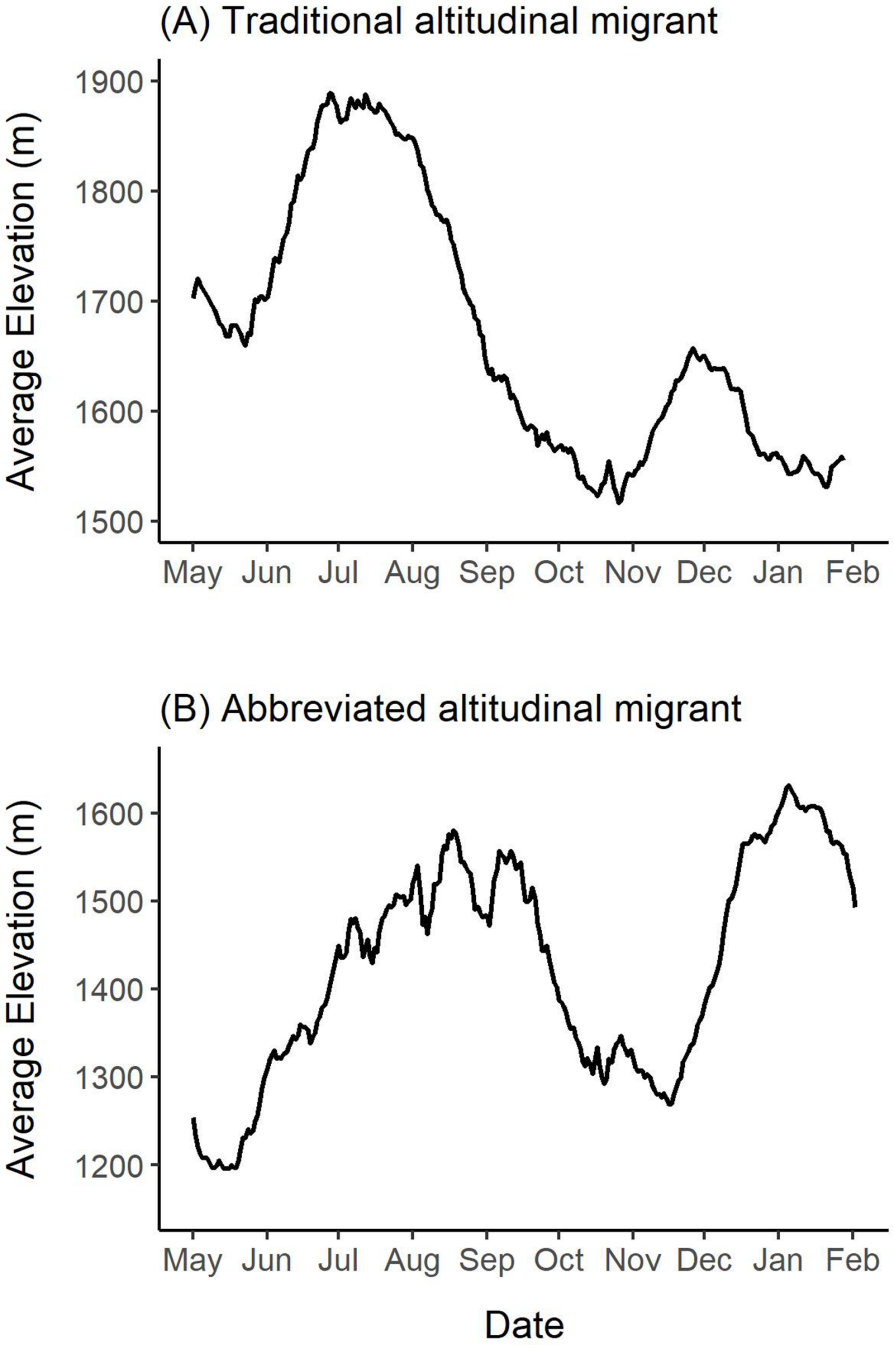
Examples of altitudinal variation exhibited by two collared female Stone’s sheep that exhibited (A) traditional altitudinal migration (female 42704) and (B) abbreviated altitudinal migration (female 41323). Mean daily elevations were calculated using a 14-day moving window from elevations extracted from GPS collar locations of females in the Cassiar Mountains, British Columbia, Canada, from May 2019–February 2020 (A; 42704) and May 2018–February 2019 (B; 41323). Ticks on the *x*-axis indicate the first day of the month.

Median elevation used by females during this study was 1,679 m ranging from 702 m to 2,282 m (Table S3). During summer, the median elevation was 1,709 m and varied from 1,563 m to 1,827 m (Table S3). In the winter, the median elevation was 1,673 m and ranged from 1,478 m to 1,751 m (Table S3). Almost all individuals (*n* = 15) occupied similar summer and winter median elevations (Δ <150 m), and thus, we concluded that they did not exhibit traditional altitudinal migration. However, one female was an exception to this, because she used a notably lower-elevation winter range with a median winter elevation of 1,531 m and a median summer elevation of 1,789 m (Δ = 257 m; female 42704 in Table S3; Fig. 6A). Figure 6 shows this female’s mean elevation use over a 14-day moving window where she used elevations in the summer that were up to 350 m higher than those used in the winter.

Many of the collared females (*n* = 14) exhibited changes in elevation use that coincide with abbreviated altitudinal migration (Fig. 6B). As we expected, almost all females (*n* = 15) descended to lower elevations in mid-April to mid-May from their winter elevations (Δ <150 m), and then gradually moved upwards over multiple weeks where they arrived at higher-elevation summer ranges in mid-July or August (Δ >150 m). Figure 6B presents an example of a female’s altitudinal migration of approximately 300 m from mid-May to August 2018. During the fall, most females (*n* = 14) descended to 14-day mean elevations that were at least 100 m lower than their summer ranges. At the beginning of the winter, the same 14 females ascended at least 100 m to higher elevations and generally remained at higher elevations over the winter (Fig. 6). One of the 16 females did not exhibit clear seasonal changes in elevation-use as she remained within the same 150 m of mean elevations throughout the entire year.

### Migration strategies

Based on our findings above, we classified 12 females from eight bands as geographic migrants (Table 2). Of the 12 geographic migrants, we further classified five females as long-distance migrants, five as short-distance migrants, and two as vacillating migrants (Table 2; Fig. 4). One female that was classified as a geographic migrant also exhibited elevation-use that coincided with traditional altitudinal migration (female 42704 described above; Fig. 6A). For simplicity, we classified this female as a geographic migrant only, but we acknowledge that traditional altitudinal migration was observed in this population. The remaining four females from two bands that were considered geographic residents, were classified as abbreviated altitudinal migrants (Table 2; Fig. 6B). As a result, we did not classify any females as residents (considered both a geographic and altitudinal resident).

## Discussion

In this study, we document for the first time a remarkable amount of variation in seasonal migration patterns among females and bands of Stone’s sheep from the same population. Some of this variability can likely be attributed to the spatial complexity of the mountainous landscapes occupied by Stone’s sheep in British Columbia.

Most of the collared population in this study migrated geographically from their winter range to a distinct summer range. The timing of spring migrations varied amongst individuals and bands (roughly two months), while the timing of fall migrations varied more substantially (roughly four months). The timing of spring migrations in ungulates has often been linked to the onset of spring vegetation growth (Sawyer & Kauffman, 2011; Monteith et al., 2011; Monteith et al., 2018; Merkle et al., 2016; Aikens et al., 2017). For example, mule deer in the Rocky Mountains were found to surf the green-wave, timing their spring migrations and use of stopover sites with the leading edge of emerging vegetation (Sawyer & Kauffman, 2011). New forage growth has high nutritional value for Stone’s sheep, and typically begins to emerge in mid-May to early July in northern British Columbia depending on altitude and spring weather conditions (Seip & Bunnell, 1985). The spring migrations observed by females aligned within the window of expected forage growth in the Cassiar Mountains (Seip, 1983). The correlations between vegetation green-up and the timing of migrations identified in ungulate populations (Sawyer & Kauffman, 2011; Merkle et al., 2016; Aikens et al., 2017) might suggest that Stone’s sheep timed their spring geographic migrations to reach summer ranges that provided access to newly emerged, highly nutritious forage. Further, we suspect that the start and end of spring migrations varied less across individuals because females likely timed their arrival on their summer range with their access to a narrow window of new forage growth. In contrast, fall migrations are not driven by the onset of plant growth, and so it may be less imperative for individuals to return to their winter range within a constricted time window to access a dwindling nutritional source. Because we did not evaluate the influence of the spring green-up on the timing of spring migrations, future research would be required to identify whether the timing of spring migrations was driven by plant phenology.

Most of the variation in migration strategies that we observed in the Cassiar Mountain population occurred across bands, rather than among individuals. We expected a lack of individual variation within bands because females from the same band are known to move and congregate together as one gregarious unit to reduce predation risk and/or as a result of matriarchal influence on range use (Berger, 1978; Krausman & Bowyer, 2003; Festa-Bianchet, 1986; Festa-Bianchet, 1988; Sabal et al., 2021). Although large bighorn sheep herds are known to exhibit partial migration (Lowrey et al., 2019), Stone’s sheep females in the Cassiar Mountains occur in smaller bands of typically five to 10 individuals, and thus we expected these small bands would remain together to reduce predation risk. Further, we expected Stone’s sheep in the same band to adopt the same seasonal movements if these movements were adapted to access seasonal resources distributed across their heterogeneous landscape. As we expected, most females that had at least one other collared female in their band exhibited the same migration strategies (*n* = 10 from five bands: Table 2). However, one band exhibited partial migration, where one individual migrated 26.5 km to a different geographic summer range, while the other two collared females used the same geographic range year-round but exhibited abbreviated migration (Band No. 4; Table 2). We recognize that we investigated the seasonal movements of only a subset of the population from one year of collar data (approximately 9% of the estimated 175 individuals). Therefore, it is possible that some Stone’s sheep from the same bands had greater variation in seasonal movements than we were able to identify in this study.

Although infrequent, some Stone’s sheep exhibited vacillating migration (as defined by Denryter, Stephenson & Monteith, 2021) between their summer and winter ranges. Our study builds on the findings from Denryter, Stephenson & Monteith (2021) that was the first to document vacillating migratory behavior in the bighorn sheep population in the Sierra Nevada Mountains, California, USA. Unlike Denryter, Stephenson & Monteith (2021), where *altitudinal* migrants vacillated between low-elevation winter range and high-elevation summer ranges, we instead found *geographic* migrants vacillated between their distinct summer and winter ranges located on different mountains over 10 km apart. Our findings demonstrate that vacillating migration is not restricted to altitudinal migrants but can be exhibited by geographic migrants as well. Further, our study builds on the understanding that vacillating migration does not occur only in the Sierra Nevada bighorn population, but also occurs in Stone’s sheep in northern British Columbia, and we suspect that it might also occur in other wild sheep populations. Vacillating migrants are expected to be more flexible in their ability to balance predator avoidance with accessing quality forage (Denryter, Stephenson & Monteith, 2021), unlike residents and migrants that typically commit to one migration strategy over an entire season, sacrificing either better forage or lower predation risk (Spitz et al., 2018). We did not assess factors influencing vacillating behaviors, but we suspect that vacillating migrants in the Cassiar Mountains adopted this strategy to realize benefits that both migrants and residents experience.

We delineated migration routes used by females and found that bands and individuals varied substantially in their distances travelled from winter to summer ranges (Fig. 5; Table 2). Geographic migrants travelled from 10 km to 47 km between their winter and summer ranges, coinciding with similar distances reported in native populations of bighorn sheep in Montana and Wyoming, USA, that travelled from roughly 5 to 55 km between seasonal ranges (Lowrey et al., 2019; Lowrey et al., 2020). It is unknown why some individuals and bands travelled farther to access summer ranges, but we suspect this could be related to the distribution of available resources and/or predation pressures across the landscape, which can influence the suitability of summer and winter ranges.

To our knowledge, little research has explored the use of stopover sites by thinhorn and bighorn sheep, although the use of stopover sites has been documented in other ungulates (Sawyer & Kauffman, 2011; Aikens et al., 2017; Paton et al., 2017; Monteith et al., 2011; Monteith et al., 2018). Stone’s sheep often used stopover sites on their migrations, and the number of stopover sites that they used varied greatly among bands (Table S1). Although we cannot determine why some bands used stopover sites while others did not, a possible explanation is that some bands may have realized a benefit to using stopover sites, such as gaining access to high-quality forage by surfing the green-wave (Aikens et al., 2017; Monteith et al., 2011; Monteith et al., 2018; Sawyer & Kauffman, 2011). However, this rationale does not explain why more individuals used stopover sites during their migrations in the fall *versus* the spring. An alternative explanation is that the timing of snowfall may impact the frequency of stopover sites used. For example, early snow in the fall or late snow melt in the spring could prevent Stone’s sheep from making stopovers. A third possible explanation is that stopover sites might function as a predator refuge for migrants travelling along their migration routes (Sabal et al., 2021). Geographic migrants are expected to experience heightened predation risk on their migrations (Bolger et al., 2008; Hebblewhite & Merrill, 2007) and this risk may increase when females are accompanied by young lambs. While migrating, sheep mostly travel through and across valley bottoms, where predators such as grizzly bears and wolves are more likely to occur (Walker et al., 2007). Further, valley bottoms generally have low visibility and limited escape terrain which are important for predator evasion in Stone’s sheep (Geist, 1971; Bleich, Bowyer & Wehausen, 1997; Koizumi & Derocher, 2019). As a result, stopover sites that provide less ‘risky’ habitats for Stone’s sheep might provide an important refuge during migrations.

Altitudinal migrants in the Cassiar Mountains used high elevations in the summer and winter, and lower elevations in the spring and fall, coinciding with the pattern of abbreviated altitudinal migration (Fig. 1; Courtemanch et al., 2017). This was unlike the seasonal movements that are often reported in bighorn sheep populations that follow traditional altitudinal migration (Spitz et al., 2018; Lowrey et al., 2020; Spitz, Hebblewhite & Stephenson, 2017). Typically, sheep occupy winter ranges at low elevations to avoid deep snow in high elevations, and then use high-elevation summer ranges once the deep snow has melted (Fig. 1). However, the use of high-elevation winter ranges, like the Stone’s sheep in this study, has been documented in some wild sheep populations as well. For example, bighorn sheep in Elk Valley in southern British Columbia (Poole et al., 2016), and Dall’s sheep in Lake Clark National Park and Preserve, Alaska (Mahoney et al., 2018; Aycrigg et al., 2021) wintered at high elevations on wind-swept slopes and ridgelines to avoid deep snow that accumulates at low elevations (Poole et al., 2016; Mahoney et al., 2018; Aycrigg et al., 2021). Wild sheep avoid deep snow because it restricts movement, inaccessibly buries forage, and affects thermoregulation (Parker, Robbins & Hanley, 1984; Dailey & Hobbs, 1989). We suspect Stone’s sheep in the Cassiar Mountains used high elevations during winter, because in the north central mountain ranges of British Columbia, lower elevations typically accumulate deep snow, while wind-blown slopes and ridges at high elevations have shallower snow cover.

Regardless of whether females migrated geographically or not, almost all females exhibited spring altitudinal migrations from lower spring elevations to higher summer ranges. In late April to late May, females descended from their high-elevation winter ranges to lower, subalpine elevations, and then gradually migrated upwards, reaching peak elevations in July and August (Fig. 6). The forage maturation hypothesis (FMH) predicts that herbivores exhibit migrations during the growing season to maximize intake of new, high-quality forage by moving in concert with plant phenology (Albon & Langvatn, 1992; Hebblewhite, Merrill & McDermid, 2008). The timing of spring altitudinal migrations observed in the Cassiar Mountain herd likely aligns with the FMH, because the timing coincides with the onset of plant growth and peak productivity in subalpine and alpine habitats (Seip, 1983; Seip & Bunnell, 1985; Albon & Langvatn, 1992). We suggest that females likely exhibited these spring altitudinal migrations to maximize access to newly emerged forage during the growing season.

We observed less significant altitudinal changes than most other populations of wild sheep that were found to exhibit strong changes in elevation (Spitz, Hebblewhite & Stephenson, 2017; Lowrey et al., 2020). This indicates that Stone’s sheep in the Cassiar Mountains are mostly associated with subalpine and alpine habitats within a narrower range of elevations and do not tend to use areas at low elevations. This could be due to high predation pressure from wolves and grizzly bears at low elevations (Sabal et al., 2021), and/or encroachment of shrubline into higher elevation areas (Melham, 2019; Dial et al., 2016; Myers-Smith & Hik, 2018).

Classifying migration strategies in mountain ungulates is challenging and rarely straightforward, because migrations often occur over horizontal and vertical planes (Lowrey et al., 2019; Lowrey et al., 2020). Often, research that aims to describe migratory behaviors do so by lumping individuals into two or three classes (*i.e.,* migratory, resident, and sometimes nomadic) based only on their movements across either geographic or altitudinal space, and fail to detect potentially unique or unusual patterns. However, recent research has highlighted the importance of describing more patterns of geographic and altitudinal migrations (Courtemanch et al., 2017; Denryter, Stephenson & Monteith, 2021; Lowrey et al., 2019). Our study took an exploratory approach that included breaking down the components of seasonal habitat use and migrations to better describe movement behaviours exhibited by individuals and bands. By doing so, we were able to identify unique movement behaviors that might otherwise have been overlooked, such as vacillating migration by geographic migrants and the use of stopover sites, which could be important findings for our understanding of migration (Fig. 4). Further, this approach allowed us to observe a diverse assemblage of migration strategies used by Stone’s sheep in the Cassiar Mountains. Our study emphasizes the importance of investigating seasonal movements over geographic *and* altitudinal space when assessing migratory behaviors and exploring individual differences.

Migratory populations benefit from having diverse migratory behaviors because these can improve long-term population sustainability and resilience to unpredictable conditions (Webster et al., 2002; Schindler et al., 2010; Lowrey et al., 2019). Lowrey et al. (2019) demonstrated that native wild sheep populations, where migrations have not been substantially altered by anthropogenic impacts (*i.e.,* highways, developments), generally exhibit the greatest migratory diversity when compared to restored or augmented populations. The Cassiar Mountain Stone’s sheep population is a native population that has not been exposed to widespread, high levels of anthropogenic disturbance, and thus, we expect that their native migrations are largely intact. As a result, the diverse assemblage of migration strategies and seasonal habitat use that was exhibited by females in this study supports Lowrey et al. (2019)’s finding that native populations with little anthropogenic exposure are more likely to display high variation in migratory behaviors. Losing native migration due to habitat loss or anthropogenic barriers (*i.e.,* highways, fencing) can diminish population stability and persistence (Whyte & Joubert, 1988; Ben-Shahar, 1993; Harris et al., 2009; Blum, Stewart & Schroeder, 2015). Because losing native migration strategies often means these strategies are lost indefinitely (Jesmer et al., 2018; Lowrey et al., 2019), it is imperative that effort be made to preserve the diverse migratory behaviors of native wild sheep (Lowrey et al., 2019; Jesmer et al., 2018). We hope our study provides insight for managers, land-use planners, and researchers to better appreciate the diversity and complexity of migratory behaviors of Stone’s sheep and to help preserve future migrations in the Cassiar Mountains.

## Conclusion

Because ungulate migrations are being lost across the globe at an unprecedented rate (Berger, 2004), it is more important than ever to document movement behaviors that individuals exhibit while migrating. Additionally, mapping the migration routes and seasonal habitats used by ungulates is an essential step in protecting future native migrations (Berger, 2004; Harris et al., 2009; Jesmer et al., 2018). By delineating seasonal ranges, migration routes, and stopover sites, we provide potential high-priority areas for Stone’s sheep that can help inform land-use planning and conservation in the region. However, the complexity in migration patterns displayed by Stone’s sheep increases the challenge of protecting the Cassiar Mountain population’s seasonal ranges and routes for migration. As such the Province of British Columbia has sought to increase citizen science contributions to help inform land management decisions and species management through the use of tools like the B.C. Mountain Goat and Wild Sheep Natal App (Government of British Columbia, 2022). Contributions of public-sourced data could help improve the overall understanding of localized migration strategies and the use of habitat features such as mineral licks, for species such as Stone’s sheep.

##  Supplemental Information

10.7717/peerj.15215/supp-1 Supplemental Information 1 Histogram of the distances travelled along migration routes between winter and summer ranges by geographically migrating collared female Stone’s sheep in the Cassiar Mountains, British Columbia, Canada, 2018–2020

10.7717/peerj.15215/supp-2 Supplemental Information 2 Start and end dates of seasonal migrations Start and end dates of spring and fall migrations, duration of migration in days, and number of stopover sites used for each collared female Stone’s sheep that exhibited a geographic migration (*n* = 12) in the Cassiar Mountains, British Columbia, Canada, 2018–2020.

10.7717/peerj.15215/supp-3 Supplemental Information 3 Start and end dates of summer and winter seasons Start and end dates of summer and winter seasons and duration (in days) for each collared female Stone’s sheep that exhibited a geographic migration (n = 12), and population median, minimum (min) and maximum (max) for all collared females in the Cassiar Mountains, British Columbia, Canada, 2018-2020.

10.7717/peerj.15215/supp-4 Supplemental Information 4 Altitudinal variation of collared female Stone’s sheep Summary of annual, winter and summer elevation use including the median (m), minimum (min; m), maximum (max; m), and altitudinal change in median elevation from winter to summer (m) for all collared female Stone’s sheep (n =16) from nine bands in the Cassiar Mountains, British Columbia, Canada, 2018-2020.

## Additional Information and Declarations

### Field Study Permissions

The following information was supplied relating to field study approvals (i.e., approving body and any reference numbers): All animal handling and capture procedures were in accordance with BC Ministry of Forests, Lands, and Natural Resource Operations protocols, and approved by the Animal Care and Use Committee at the University of Alberta (no. AUP00002992).

### Data Availability

The following information was supplied regarding data availability: The raw data is owned by the Government of British Columbia and access is restricted due to the sensitive nature of the data. Requests to access the data should be submitted at: https://www2.gov.bc.ca/gov/content/environment/plants-animals-ecosystems/conservation-data-centre/request-cdc-data.
