# Supplementary figures and images for: Diverse migration patterns and seasonal habitat use of Stone’s sheep (Ovis dalli stonei)
Source: PeerJ. 2023 Jun 16;11:e15215. doi: 10.7717/peerj.15215 (PMC10278595; doi:10.7717/peerj.15215)

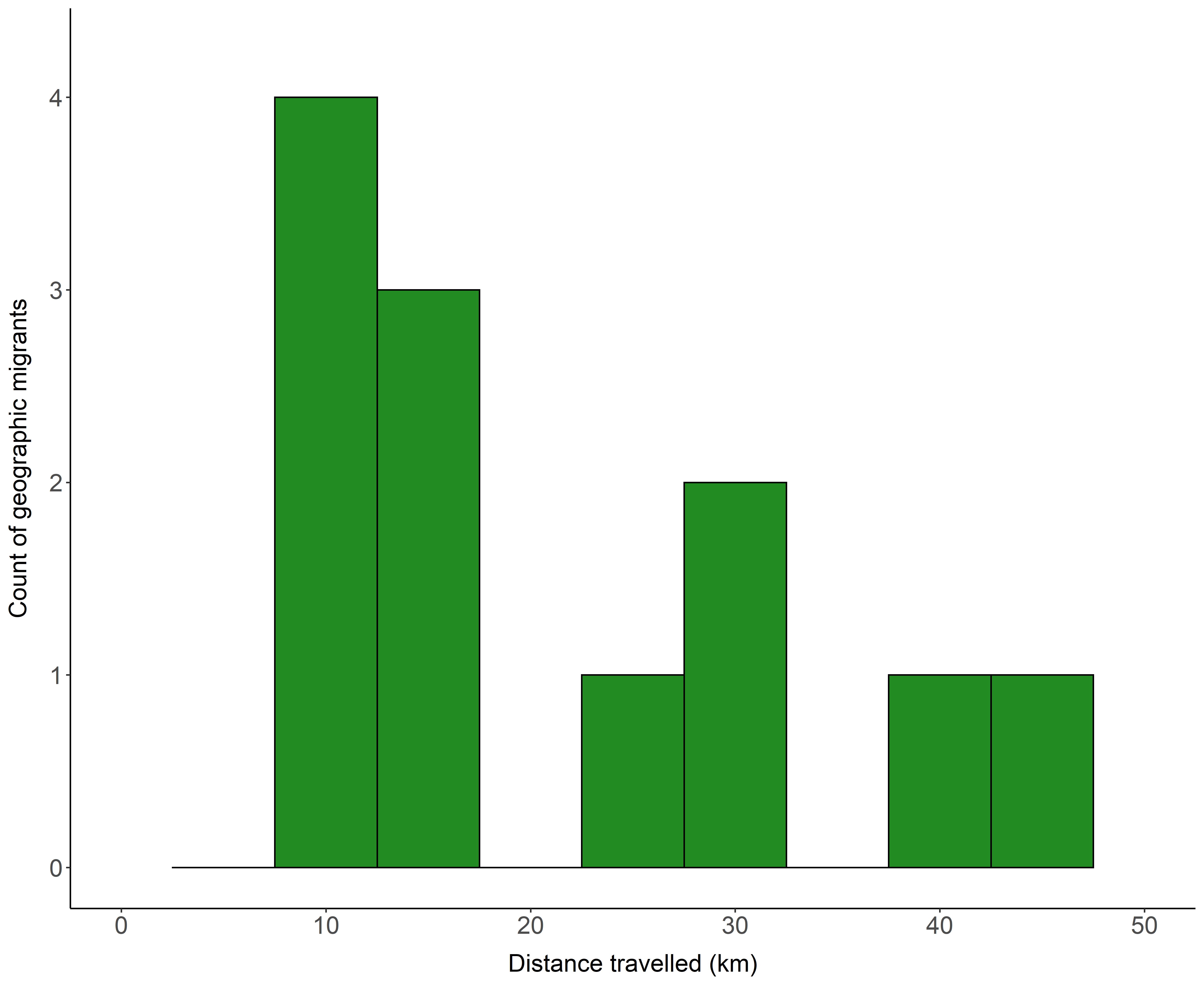

Supplement: Supplemental Information 1 [file peerj-11-15215-s001.jpeg]
