# Supplementary material for: Diverse migration patterns and seasonal habitat use of Stone’s sheep (Ovis dalli stonei)
Source: PeerJ. 2023 Jun 16;11:e15215. doi: 10.7717/peerj.15215 (PMC10278595; doi:10.7717/peerj.15215)
Supplement: Supplemental Information 2 — Start and end dates of spring and fall migrations, duration of migration in days, and number of stopover sites used for each collared female Stone’s sheep that exhibited a geographic migration (n = 12) in the Cassiar Mountains, British Columbia, Canada, 2018–2020. [file peerj-11-15215-s002.docx]

| **Band No.** | ***n*** | **Female ID** | **Year** | **Spring migration** | | |  |  | **Fall migration** | | |  |
| --- | --- | --- | --- | --- | --- | --- | --- | --- | --- | --- | --- | --- |
|  |  |  |  | Start date | End date | Duration (days) | Stopover sites used |  | Start date | End date | Duration (days) | Stopover sites used |
| 1 | 1 | 42696 | 2019 | 2019-06-25 | 2019-07-04 | 9 | 3 |  | 2019-08-16 | 2019-10-14 | 59 | 6 |
|  |  |  |  |  |  |  |  |  |  |  |  |  |
| 2 | 1 | 42703 | 2019 | 2019-06-12 | 2019-06-17 | 5 | 4 |  | 2019-09-21 | 2019-09-24 | 3 | 1 |
|  |  |  |  |  |  |  |  |  |  |  |  |  |
| 3 | 2 | 42701 | 2019 | 2019-06-29 | 2019-07-01 | 2 | 0 |  | 2019-09-09 | 2019-09-21 | 12 | 5 |
|  |  | 42704 | 2019 | 2019-06-26 | 2019-06-27 | 1 | 0 |  | 2019-08-20 | 2019-09-01 | 12 | 4 |
|  |  |  |  |  |  |  |  |  |  |  |  |  |
| 4 | 3 | 42698 | 2019 | 2019-07-10 | 2019-08-05 | 26 | 3 |  | 2019-09-19 | 2019-10-03 | 14 | 2 |
|  |  | 41320 | 2019 | - | - | - | - |  | - | - | - | - |
|  |  | 42702 | 2019 | - | - | - | - |  | - | - | - | - |
|  |  |  |  |  |  |  |  |  |  |  |  |  |
| 5 | 1 | 41324 | 2019 | 2019-05-25 | 2019-05-28 | 3 | 1 |  | 2019-10-08 | 2019-10-11 | 3 | 1 |
|  |  |  |  |  |  |  |  |  |  |  |  |  |
| 6 | 2 | 41318 | 2019 | 2019-06-05 | 2019-06-17 | 12 | 2 |  | 2019-08-21 | 2019-08-24 | 3 | 1 |
|  |  | 42699 | 2019 | 2019-06-05 | 2019-06-26 | 21 | 4 |  | 2019-08-17 | 2019-08-23 | 6 | 2 |
|  |  |  |  |  |  |  |  |  |  |  |  |  |
| 7 | 2 | 41321 | 2018 | 2018-05-20 | 2018-06-14 | 25 | 2 |  | 2018-08-21 | 2018-08-23 | 2 | 0 |
|  |  | 41323 | 2018 | 2018-06-26 | 2018-07-05 | 9 | 1 |  | 2018-09-27 | 2018-10-31 | 34 | 3 |
|  |  |  |  |  |  |  |  |  |  |  |  |  |
| 8 | 2 | 42695 | 2019 | 2019-06-12 | 2019-06-13 | 1 | 0 |  | 2019-12-11 | 2020-01-07 | 27 | 5 |
|  |  | 42697 | 2019 | 2019-06-10 | 2019-06-11 | 1 | 0 |  | 2019-12-11 | 2020-01-07 | 27 | 5 |
|  |  |  |  |  |  |  |  |  |  |  |  |  |
| 9 | 2 | 41322 | 2019 | - | - | - | - |  | - | - | - | - |
|  |  | 42700 | 2019 | - | - | - | - |  | - | - | - | - |
|  |  |  |  |  |  |  |  |  |  |  |  |  |
|  | | **Total median** | | 12-Jun | 17-Jun | 7 | 1.5 |  | 1.5 | 22-Sep | 12 | 2.5 |
|  |  | **Total min** | | 20-May | 14-Jun | 1 | 0 |  | 0 | 23-Aug | 2 | 0 |
|  |  | **Total max** | | 10-Jul | 05-Aug | 26 | 4 |  | 4 | 07-Jan | 59 | 6 |

- = not applicable.
