# Supplementary material for: Diverse migration patterns and seasonal habitat use of Stone’s sheep (Ovis dalli stonei)
Source: PeerJ. 2023 Jun 16;11:e15215. doi: 10.7717/peerj.15215 (PMC10278595; doi:10.7717/peerj.15215)
Supplement: Supplemental Information 3 — Start and end dates of summer and winter seasons and duration (in days) for each collared female Stone’s sheep that exhibited a geographic migration (n = 12), and population median, minimum (min) and maximum (max) for all collared females in the Cassiar Mountains, British Columbia, Canada, 2018-2020. [file peerj-11-15215-s003.docx]

| **Band No.** | ***n*** | **Female ID** | **Year** | **Winter** | | |  | **Summer** | | |
| --- | --- | --- | --- | --- | --- | --- | --- | --- | --- | --- |
|  |  |  |  | Start date | End date | Duration (days) |  | Start date | End date | Duration (days) |
| 1 | 1 | 42696 | 2019 | 2019-10-14 | 2019-06-25 | 111 |  | 2019-07-04 | 2019-08-16 | 43 |
| 2 | 1 | 42703 | 2019 | 2019-09-24 | 2019-06-12 | 104 |  | 2019-06-17 | 2019-09-21 | 96 |
| 3 | 2 | 42701 | 2019 | 2019-09-21 | 2019-06-29 | 84 |  | 2019-07-01 | 2019-09-09 | 70 |
|  |  | 42704 | 2019 | 2019-09-01 | 2019-06-26 | 67 |  | 2019-06-27 | 2019-08-20 | 54 |
| 4 | 3 | 42698 | 2019 | 2019-10-03 | 2019-07-10 | 85 |  | 2019-08-05 | 2019-09-19 | 45 |
|  |  | 41320 | 2019 | - | - | - |  | - | - | - |
|  |  | 42702 | 2019 | - | - | - |  | - | - | - |
| 5 | 1 | 41324 | 2019 | 2019-10-11 | 2019-05-25 | 139 |  | 2019-05-28 | 2019-10-08 | 133 |
| 6 | 2 | 41318 | 2019 | 2019-08-24 | 2019-06-05 | 80 |  | 2019-06-17 | 2019-08-21 | 65 |
|  |  | 42699 | 2019 | 2019-08-23 | 2019-06-05 | 79 |  | 2019-06-26 | 2019-08-17 | 52 |
| 7 | 2 | 41321 | 2018 | 2018-08-23 | 2018-05-20 | 95 |  | 2018-06-14 | 2018-08-21 | 68 |
|  |  | 41323 | 2018 | 2018-10-31 | 2018-06-26 | 127 |  | 2018-07-05 | 2018-09-27 | 84 |
| 8 | 2 | 42695 | 2019 | 2020-01-07 | 2019-06-12 | 209 |  | 2019-06-13 | 2019-12-11 | 181 |
|  |  | 42697 | 2019 | 2020-01-07 | 2019-06-10 | 211 |  | 2019-06-11 | 2019-12-11 | 183 |
| 9 | 2 | 41322 | 2019 | - | - | - |  | - | - | - |
|  |  | 42700 | 2019 | - | - | - |  | - | - | - |
|  | | **Total median** | | 01-Sep | 05-Jun | 99.5 |  | 13-Jun | 21-Aug | 69 |
|  |  | **Total min** | | 23-Aug | 20-May | 67 |  | 14-Jun | 21-Aug | 43 |
|  |  | **Total max** | | 07-Jan | 10-Jul | 211 |  | 05-Aug | 11-Dec | 183 |

- = not applicable.
