# Supplementary material for: Diverse migration patterns and seasonal habitat use of Stone’s sheep (Ovis dalli stonei)
Source: PeerJ. 2023 Jun 16;11:e15215. doi: 10.7717/peerj.15215 (PMC10278595; doi:10.7717/peerj.15215)
Supplement: Supplemental Information 4 — Summary of annual, winter and summer elevation use including the median (m), minimum (min; m), maximum (max; m), and altitudinal change in median elevation from winter to summer (m) for all collared female Stone’s sheep (n =16) from nine bands in the Cassiar Mountains, British Columbia, Canada, 2018-2020. [file peerj-11-15215-s004.docx]

| **Band No.** | ***n*** | **Female ID** | **Year** |  | **Annual** | | |  | **Winter** | | |  | **Summer** | | |  | **Altitudinal Variation^a^** |
| --- | --- | --- | --- | --- | --- | --- | --- | --- | --- | --- | --- | --- | --- | --- | --- | --- | --- |
|  |  |  |  |  | **Median** | **Min** | **Max** |  | **Median** | **Min** | **Max** |  | **Median** | **Min** | **Max** |  |  |
| 1 | 1 | 42696 | 2019 |  | 1718.1 | 907.5 | 2125.1 |  | 1708.8 | 1245.2 | 2091.7 |  | 1752.8 | 1354.7 | 2069.3 |  | -44.0 |
|  |  |  |  |  |  |  |  |  |  |  |  |  |  |  |  |  |  |
| 2 | 1 | 42703 | 2019 |  | 1715.2 | 1212.6 | 2168.9 |  | 1653.5 | 1291.8 | 2168.9 |  | 1685.1 | 1356.9 | 2076.0 |  | -31.6 |
|  |  |  |  |  |  |  |  |  |  |  |  |  |  |  |  |  |  |
| 3 | 2 | 42704 | 2019 |  | 1676.8 | 1177.8 | 2133.5 |  | 1531.7 | 1488.7 | 2066.4 |  | 1788.8 | 1178.0 | 2026.1 |  | -257.1 |
|  |  | 42701 | 2019 |  | 1770.6 | 1165.0 | 2133.5 |  | 1819.6 | 1423.4 | 2072.5 |  | 1827.3 | 1418.3 | 2029.5 |  | -7.7 |
|  |  |  |  |  |  |  |  |  |  |  |  |  |  |  |  |  |  |
| 4 | 3 | 42698 | 2019 |  | 1578.8 | 814.7 | 2139.2 |  | 1599.1 | 1107.8 | 1785.2 |  | 1709.3 | 1373.0 | 2139.2 |  | -110.2 |
|  |  | 41320 | 2019 |  | 1496.3 | 808.6 | 1862.3 |  | 1478.6 | 1099.9 | 1811.1 |  | 1600.3 | 834.0 | 1848.6 |  | -121.7 |
|  |  | 42702 | 2019 |  | 1542.0 | 820.6 | 1863.8 |  | 1518.1 | 1175.3 | 1789.7 |  | 1563.2 | 1031.1 | 1840.8 |  | -45.1 |
|  |  |  |  |  |  |  |  |  |  |  |  |  |  |  |  |  |  |
| 5 | 1 | 41324 | 2019 |  | 1794.8 | 1351.1 | 2281.5 |  | 1802.9 | 1430.8 | 2122.2 |  | 1809.7 | 1405.0 | 2173.4 |  | -6.8 |
|  |  |  |  |  |  |  |  |  |  |  |  |  |  |  |  |  |  |
| 6 | 2 | 41318 | 2019 |  | 1735.0 | 784.6 | 2139.1 |  | 1709.2 | 1308.8 | 2087.6 |  | 1752.6 | 1389.5 | 2105.2 |  | -43.4 |
|  |  | 42699 | 2019 |  | 1722.4 | 797.4 | 2164.8 |  | 1731.4 | 1356.0 | 2145.9 |  | 1710.0 | 1405.4 | 1993.0 |  | 21.4 |
|  |  |  |  |  |  |  |  |  |  |  |  |  |  |  |  |  |  |
| 7 | 2 | 41321 | 2018 |  | 1651.9 | 702.2 | 1988.3 |  | 1688.1 | 1396.7 | 1852.0 |  | 1625.2 | 769.8 | 1865.0 |  | 62.9 |
|  |  | 41323 | 2018 |  | 1501.6 | 769.8 | 1988.3 |  | 1589.5 | 830.0 | 1787.9 |  | 1633.8 | 769.8 | 1939.3 |  | -44.3 |
|  |  |  |  |  |  |  |  |  |  |  |  |  |  |  |  |  |  |
| 8 | 2 | 42697 | 2019 |  | 1759.8 | 1398.1 | 2214.6 |  | 1769.2 | 1461.3 | 2035.3 |  | 1764.3 | 1405.0 | 2069.6 |  | 4.9 |
|  |  | 42695 | 2019 |  | 1761.0 | 1241.6 | 2132.5 |  | 1776.1 | 1465.0 | 2027.2 |  | 1769.5 | 1396.5 | 2069.6 |  | 6.6 |
|  |  |  |  |  |  |  |  |  |  |  |  |  |  |  |  |  |  |
| 9 | 2 | 41322 | 2019 |  | 1622.1 | 766.0 | 1966.4 |  | 1674.2 | 773.6 | 1925.8 |  | 1666.7 | 766.0 | 1937.7 |  | 7.5 |
|  |  | 42700 | 2019 |  | 1640.9 | 794.9 | 1981.6 |  | 1649.5 | 800.5 | 1909.4 |  | 1671.8 | 1143.0 | 1981.6 |  | -22.3 |

^a^Altitudinal variation was calculated as the difference in median elevation used in winter versus summer. A negative value indicates use of a higher median elevation in the summer compared to winter.
